# Co-Stimulation through 4-1BB/CD137 Improves the Expansion and Function of CD8^+^ Melanoma Tumor-Infiltrating Lymphocytes for Adoptive T-Cell Therapy

**DOI:** 10.1371/journal.pone.0060031

**Published:** 2013-04-01

**Authors:** Jessica Ann Chacon, Richard C. Wu, Pariya Sukhumalchandra, Jeffrey J. Molldrem, Amod Sarnaik, Shari Pilon-Thomas, Jeffrey Weber, Patrick Hwu, Laszlo Radvanyi

**Affiliations:** 1 Department of Melanoma Medical Oncology, The University of Texas M. D. Anderson Cancer Center, Houston, Texas, United States of America; 2 The Immunology Program of the University of Texas Health Science Center, Graduate School of Biomedical Sciences, The University of Texas M. D. Anderson Cancer Center, Houston, Texas, United States of America; 3 Department of Stem Cell Transplantation, University of Texas, M. D. Anderson Cancer Center, Houston, Texas, United States of America; 4 Donald A. Adam Comprehensive Melanoma Research Center, Moffitt Cancer Center, Tampa, Florida, United States of America; Maisonneuve-Rosemont Hospital, Canada

## Abstract

Adoptive T-cell therapy (ACT) using tumor-infiltrating lymphocytes (TIL) can induce tumor regression in up to 50% or more of patients with unresectable metastatic melanoma. However, current methods to expand melanoma TIL, especially the “rapid expansion protocol” (REP) were not designed to enhance the generation of optimal effector-memory CD8^+^ T cells for infusion. One approach to this problem is to manipulate specific co-stimulatory signaling pathways to enhance CD8^+^ effector-memory T-cell expansion. In this study, we determined the effects of activating the TNF-R family member 4-1BB/CD137, specifically induced in activated CD8^+^ T cells, on the yield, phenotype, and functional activity of expanded CD8^+^ T cells during the REP. We found that CD8^+^ TIL up-regulate 4-1BB expression early during the REP after initial TCR stimulation, but neither the PBMC feeder cells in the REP or the activated TIL expressed 4-1BB ligand. However, addition of an exogenous agonistic anti-4-1BB IgG_4_ (BMS 663513) to the REP significantly enhanced the frequency and total yield of CD8^+^ T cells as well as their maintenance of CD28 and increased their anti-tumor CTL activity. Gene expression analysis found an increase in bcl-2 and survivin expression induced by 4-1BB that was associated with an enhanced survival capability of CD8^+^ post-REP TIL when re-cultured in the absence or presence of cytokines. Our findings suggest that adding an agonistic anti-4-1BB antibody during the time of TIL REP initiation produces a CD8^+^ T cell population capable of improved effector function and survival. This may greatly improve TIL persistence and anti-tumor activity *in vivo* after adoptive transfer into patients.

## Introduction

Adoptive transfer of *ex vivo* expanded autologous tumor-infiltrating lymphocytes together followed by one to two cycles of high-dose IL-2 therapy has emerged in multiple Phase II clinical trials to be one of the most powerful therapies for unresectable metastatic melanoma [1]–[4]. Durable clinical response rates of up to 50% have been consistently reported using a current protocol consisting of a lymphodepleting preconditioning regimen using cyclophosphamide and fludaribine followed by expanded TIL infusion and IL-2. Our group at MD Anderson Cancer Center (MDACC) has recently completed a study on 31 metastatic patients that have failed multiple first- and second- line therapies using this regimen and reported a 48% clinical response rate [3]. Most responding patients have had progression-free survival times after treatment of >15 months, which is longer than those achieved using other therapies, including targeted therapies with MAPK inhibitors [5]. Although other forms of T-cell therapy (e.g., TCR- and CAR- transduced T cells) have become available [6], TIL therapy has still remained the superior form of therapy for melanoma because it targets many tumor antigens recognized by a more heterogenous population of T cells rather than a single antigen that can be lost due to the high mutation rates in melanomas [7].

One of the key issues in TIL therapy when determining whether objective tumor regression will occur is the phenotype of the T cells infused and their persistence *in vivo* following adoptive transfer. Melanoma TIL consists mostly of activated TCRαβ^+^ CD4^+^ and CD8^+^ T cells with heterogeneous phenotypes ranging from less differentiated effector-memory cells to more differentiated cells that have lost critical co-stimulatory molecules, such as CD27 and CD28 [8], [9]. Recent data from our group and others has found that higher frequencies and total numbers of infused effector-memory CD8^+^ T cells correlated highly with clinical response suggesting that CD8^+^ T cells in the TIL infusion product are the most critical T cells mediating objective tumor regression [1], [3], [10]. Other studies have found that expanded CD8^+^ TIL maintaining CD28 expression and other effector-memory phenotypic markers, such as CD27, are associated with longer telomere length and persist longer *in vivo* after adoptive transfer [11], [12]. Thus, accumulating evidence indicates that higher numbers and frequencies of CD8^+^ T cells maintaining effector-memory properties (e.g., CD28 expression) for enhanced survival together with the ability to induce cytolytic molecules, such as perforin and granzyme B, for tumor killing would be the optimal cells to generate for melanoma TIL therapy.

The current method to generate the final TIL product for infusion uses a “rapid expansion protocol” (REP) consisting of taking TIL initially expanded from tumor fragments with IL-2 alone for 3–4 weeks and activating them with anti-CD3 in the presence of a large excess (200∶1 ratio) of irradiated PBMC feeder cells [13]. The cells are then expanded for 2 weeks by feeding with culture medium and IL-2. The feeder cells presumably provide a source of Fc receptors for anti-CD3 cross-linking as well as some limited growth factors, anti-oxidants, and co-stimulatory factors for TIL expansion [13]. However, the current REP protocol, is not really geared towards optimizing the yield of highly functional effector-memory CD8^+^ T cells and many times results in a loss of CD8^+^ T cells due to the over-growth of CD4^+^ T cells in the final product. Furthermore, the remaining population of CD8^+^ T cells have lost CD28 expression and have sub-optimal levels of cytolytic granule molecule expression [8], [9], [14]. These CD8^+^ T cells can be susceptible to cell death and can be hypo-responsive to re-stimulation with melanoma antigens [8], [14].

Previously, we showed that post-REP CD8^+^ TIL that have lost CD28 and CD27 expression retained the capacity to induce “alternative” co-stimulatory molecules of the TNF-R family, especially 4-1BB/CD137 [14]. Re-stimulation of post-REP TIL using anti-CD3 or with melanoma cells resulted in activation-induced cell death (AICD) that could be potently prevented using agonistic anti-4-1BB antibodies [14]. The 4-1BB costimulated post-REP CD8^+^ TIL could also expand further with IL-2, exhibited enhanced ability to kill tumor targets, and expressed increased bcl-2 expression. In this study, we tested the effects of enhanced 4-1BB co-stimulation earlier in the process by determining its effects at the beginning of the TIL REP on the yield of CD8^+^ T cells after expansion and their phenotypic and functional properties. We found that CD8^+^ TIL up-regulate 4-1BB during the first few days of initiating the REP and provision of an agonistic fully human anti-4-1BB monoclonal antibody (mAb) enhanced the yield of CD8^+^ T cells as well as maintained effector-memory properties post-REP. In addition, MART-1 peptide-specific post-REP CD8^+^ T cells that had received 4-1BB co-stimulation at the start of the REP were more responsive to antigenic re-stimulation with mature dendritic cells pulsed with MART-1 peptide.

## Materials and Methods

### Reagents

A fully human and purified IgG4 monoclonal antibody (mAb) against human CD137 (BMS-663513; Lot 6A20377) was kindly provided by Bristol Myers Squibb (BMS; Princeton, NJ) through a Materials Transfer Agreement [15]. BMS-663513 (henceforth called anti-4-1BB) was stored at 4°C at a concentration of 14.9 mg/ml and was certified to have <0.5 EU/mg endotoxin level, >95% purity, and <5% high molecular weight species. Human recombinant IL-2 (Proleukin™) was generously provided by Prometheus Therapeutics and Diagnostics (San Diego, CA). Flow cytometry reagents were obtained from BD Biosciences, eBioscience, or BioLegend.

### Isolation and Expansion of TIL from Human Melanoma Patient Tumors

The tumor samples from metastatic lesions isolated during palliative surgery at MD Anderson Cancer Center were obtained using an Institutional Review Board (IRB) approved laboratory protocol (LAB06-0755). The tumor samples were cut into 3–5 mm^2^ fragments and placed in TIL culture media (TIL-CM) and 6,000 IU/ml IL-2 (Proleukin™) in 24-well plates for a period of 4–5 weeks (1 fragment per well). The TIL-CM contained RPMI 1640 with Glutamax (Gibco/Invitrogen; Carlsbad, CA), 1 mM pyruvate (Gibco/Invitrogen; Carlsbad, CA), 20 µg/ml Gentamicin (Gibco/Invitrogen; Carlsbad, CA), 50 µM 2-mercaptoethanol (Gibco/Invitrogen; Carlsbad, CA), 10% human AB serum (Sigma-Aldrich, St. Louis, MO) and 1X Pen-Strep (Gibco/Invitrogen; Carlsbad, CA). The TIL-CM was used for the rest of our experiments. The TIL were split 1∶1 in new TIL-CM and IL-2 across the plate from well-to-well after reaching confluence. After 4–5 weeks, the cells were harvested and designated as “pre-rapid expansion protocol TIL” (pre-REP TIL). Pre-REP TIL that were not expanded immediately further expanded using the REP were cryopreserved in 10% DMSO, 90% human AB serum and stored in liquid nitrogen.

### Rapid Expansion Protocol (REP)

The REP was performed in upright T-25 flasks by activating 1.3×10^5^ pre-REP TIL with 26×10^6^ allogeneic, irradiated (5,000cGy) PBMC feeder cells with 30 ng/ml OKT3 (anti-CD3; Abbott Labs, Abbott Park, IL) in 1∶1 mixture of TIL-CM and AIM-V (Invitrogen). Exogenous anti-4-1BB was added to some of the flasks at different concentrations on day 0 of the REP. On day 2 of the REP, 6,000 IU/ml IL-2 was added to each flask. The TIL were expanded for another 12 days. On days 5, 7, 9, and 12 of the REP, the TIL were diluted as necessary with a 1∶1 ratio of TIL-CM and AIM-V with a final concentration of 6,000 IU/ml IL-2 to keep the cells between 1–2×10^6^/ml.

### Flow Cytometric Analysis of TIL

Pre-REP and post-REP TIL were washed with FACS Wash Buffer (FWB) that contained Dulbecco’s Phosphate Buffered Saline 1X (D-PBS; Gibco/Invitrogen) and 1% Bovine Serum Albumin (BSA). The cells were resuspended in 0.1 ml FACS Staining Buffer (FSB) consisting of 1X D-PBS, 1% BSA, and 5% goat serum and stained on ice for 20 min using fluorochrome-conjugated monoclonal antibodies recognizing the following surface and intracellular markers: CD8, CD4, CD27, CD28, KLRG-1, Eomesodermin, Granzyme B (GB), and Perforin (Perf). The TIL were washed with FWB and re-suspended in 0.3 ml 1X D-PBS, 1% para-formaldehyde solution. The stained cells were analyzed using the BD FACScanto II flow cytometry analyzer using FACSDiva software. The data was later analyzed using FlowJo software (TreeStar).

### Analysis of 4-1BB and 4-1BBL Expression during the REP

Pre-REP TIL were labeled with 1 µM CFSE (Molecular Probes-Invitrogen, Carlsbad, CA) in order to be able to distinguish the TIL from the irradiated PBMC. The REP was then set-up as described above. On day 1 and day 2, the cells were harvested from the REP and stained for CD8, CD3, 4-1BB, and 4-1BBL (BD Biosciences). Gating was done on the viable cell population and then on the CFSE-positive (TIL) or CFSE-negative (feeders) population. The different populations were then analyzed for their expression of 4-1BB and 4-1BBL.

### Flow Cytometric Sorting of Post-REP CD8^+^ TIL

Post-REP TIL were harvested, washed, and re-suspended at 25×10^6^/ml in 2 ml of sterile FSB. The cells were stained using anti-CD8-Pacific Blue on ice for 20 minutes, washed, re-suspended in sterile FWB, and the CD8^+^ subset isolated by sorting in a FACSAria sorter (BD Biosciences, San Jose, CA). The sorted cells were washed in cold FWB and rested for 3 hours in order to shed the antibody. Afterwards, RNA was isolated from the sorted TIL for quantitative real-time PCR (qRT-PCR) analysis and/or cytotoxic T-cell assay.

### Quantitative Real-time PCR

We used quantitative real-time PCR (qRT-PCR) to measure the expression of anti-apoptotic genes Bcl-2 and Bcl-xL or pro-apoptotic gene Bim. RNA was isolated from 5×10^6^ post-cells on day 14 of the REP (post-REP cells) using the Qiagen RNeasy Mini Kit (Qiagen; Duesseldorf, Germany). By this time no remaining irradiated PBMC feeder cells were left in the cultures [13]. RNA quantity and was determined using a NanoDrop spectrophotometer (Thermo Scientific; Wilmington, DE). The RNA was then subjected to qRT-PCR analysis as previously described [14]. The following primer sequences were used; *bcl-2*: forward primer: 5′-CAGAAGGGACTGAATCGGAG-3′, reverse primer: 5′-TGGGATGTCAGGTCA CTGAA-3′; *bcl-x_L_*: forward primer: 5′-TGAGTCGGATCGCAGC TTGG-3′, reverse primer: 5′-TGGATGGTCAGTGTCTGGTC-3′; *bim*: forward primer: 5-ACAGGAGCC CAGCACCCATG-3′, reverse primer: 5′-ACGCCGCAACTCTTGGGCGA-3′; and *β-actin*: forward primer: 5′-TTGCCGACAGGATGCAGAA-3′, reverse primer: 5′ GCCGATCCACACGGAGTACT-3′.

### Cytotoxic T-cell Assay

TIL from HLA-A0201^+^ patients having a significant pre-REP CD8^+^ T-cell population recognizing the Melan-A/MART-1 peptide (ELAGIGILTV), as determined using HLA-A0201-peptide tetramer staining, were subjected to the REP with or without added anti-4-1BB on day 0. The post-REP cells were sorted for CD8^+^ T cells and evaluated for their cytolytic function using a caspase-3 cleavage CTL assay as previously described [16]. The target cells included HLA-A0201^+^624 melanoma cells or MART-1 peptide-pulsed T2 human lymphoma cells. The HLA-A unmatched melanoma cell line 938 and T2 cells pulsed with a human immunodeficiency virus rev peptide (ILKEPVHGV) were used as controls. The target cells were labeled with DDAO-SE (Molecule Probes-Invitrogen) for 15 min at 37°C. The sorted TIL and target cells were incubated at different effector-to-target ratios, as indicated for 3–4 h at 37°C before harvesting and staining for cleaved caspase 3 in the target cells [16].

### Multiplex Cytokine Assay

Post-REP TIL were harvested and washed twice in TIL-CM to remove any excess IL-2 and plated for 24 h in a 96 well plates pre-coated with or without 10 ng/ml anti-CD3. The supernatants were collected and cytokine secretion was measured using a Luminex-100 system using beads recognizing IL-2, IFN-γ, and TNF-α, (Millipore, Billerica, MA). The net cytokine levels after subtraction of control wells without anti-CD3 were determined.

### Cell Survival Assays Post-REP

Post-REP TIL were harvested, washed twice in TIL-CM, and re-cultured in a 24 well plates at 400,000 cells per well in 2-ml TIL-CM with no cytokine or 200 IU/ml IL-2. After 4 days, the cells were harvested and stained with anti-CD8, 7-AAD, and Annexin-V, as described previously [8], [14]. The stained cells were acquired using a FACSCanto II flow cytometer. The samples were analyzed by first gating on the live population. The percentage of CD8^+^ cells and AnnexinV^+^ was determined. Viable cell counts were performed using a hemocytometer and used to calculate the total number of live CD8^+^ T cells in the cultures using the percentage of live CD8^+^ T cells found by flow cytometry.

### Re-stimulation of Post-REP TIL with MART-1 Peptide-pulsed Dendritic Cells

Post-REP TIL from HLA-A0201^+^ patients having at least 0.5% of the CD8^+^ T cells staining positive for MART-1 tetramer (ELAGIGILTV) peptide were re-stimulated for 7 days with mature HLA-A0201-matched DC pulsed with the same MART-1 peptide (ELAGIGILTV), as described previously [8]. Dendritic cells (DCs) were generated from adherent monocytes from HLA-A0201^+^ normal donors using GM-CSF and IL-4 for 5 days following by incubation with IL-1β, TNF-α, IL-6, and PGE_2_ for 2 days [8]. The mature DCs were isolated, pulsed with MART-1 peptide for 90 min and then washed. Post-REP TIL were isolated and washed in TIL-CM two times and rested in TIL-CM for 6 hours without any additional cytokine. The cells were labeled with the cell division dye eFluor670 (Invitrogen) according to the manufacturer’s instructions and washed. The labeled TIL and peptide-pulsed DCs were mixed in 24-wells at a 10∶1 ratio (2×10^6^ TIL plus 0.2×10^6^ DCs). IL-2 (100 IU/ml) was added to all cultures to facilitate TIL viability. After 7 days, the cells were stained for CD8 and MART-1 tetramer and analyzed for cell division using a FACSCanto II (BD Biosciences).

### Polymerase Chain Reaction (PCR)

PCR was performed using a panel of optimized primers specific for 24 members of the TCRVβ family. Briefly, RNA was extracted from 1×10^6^ pre-REP TIL using Trizol (Invitrogen, Carlsbad, CA) according to the manufacturer’s instructions. The TIL then underwent the REP with or without the addition of anti-4-1BB. RNA was then isolated the same way on the post-REP TIL. The extracted RNA (1 µg) was treated with DNAse (Ambion, Austin, TX) to remove contaminating genomic DNA. All of the DNAse-treated RNA was used to synthesize cDNA by reverse transcription using the manufacturer’s protocol with the SuperScript™ III Reverse Transcriptase (Invitrogen). PCR was then performed by combining 0.5 µM of one Vβ primer for each of the different TCRVβ families with 0.5 µM of a Cβ primer, which was used for each of the 24 reactions. The PCR products were visualized on an 1.5% agarose gel.

### TCR Vβ Spectratype Analysis

For TCR Vβ spectratype analysis, PCR products were diluted in nuclease-free water so that 1.5 ng of the PCR product from each TCR Vβ family was subjected to capillary electrophoresis using an OpenGene™ System (Bayer, Terrytown, NY). Because the positions of the 5′Vβ and the 3′Cβ primers are fixed, variation in length of the PCR fragments within any TCR Vβ family is due to differences in length of the CDR3 regions. Data are presented as fluorescence intensity versus DNA fragment length. The TCR Vβ10 and Vβ19 families are pseudogenes and were therefore excluded from analysis. The number of CDR3 sequence peaks were plotted for each major Vβ family.

### Statistical Analysis

Statistical analysis for comparison of 2 groups was done using the Wilcoxon signed rank test or Student’s t test (paired datasets), or the Wilcoxon rank sum test (unpaired datasets). Analysis of experiments with 3 or more treatment groups was done using the one-way or two-way analysis of variance (ANOVA), with Bonferroni post-tests with both tests using biological relevance occurring when p<0.05. Statistical analysis was done using Graph Pad Prism (La Jolla, CA).

## Results

### Induction of 4-1BB Expression without Induction of 4-1BB Ligand after Initiation of the REP

We were first interested to determine whether 4-1BB is induced on CD8^+^ T cells early during the REP and whether the PBMC feeder cells or the TIL themselves express appreciable levels of the ligand for 4-1BB (4-1BBL) as a possible endogenous source of 4-1BB co-stimulation for the CD8^+^ T cells. For these experiments TIL were labeled with CFSE to distinguish them from the feeder cells before being activated by anti-CD3 in the REP. 4-1BB is up-regulated on T cells 24–48 hours after activation [14], [17]. Thus, we analyzed CD8, 4-1BB and 4-1BBL expression on the live TIL (CFSE^+^) and feeder cells (CFSE^-^) 1–2 days after REP initiation. Live cells were gated and the CD8^+^CFSE^+^ population and the live CFSE^−^ lymphocyte population analyzed for 4-1BB and 4-1BBL expression. We found that in each case a significant frequency of the CD8^+^ T cells in the TIL (40%-60%) had induced 4-1BB expression relative to their corresponding pre-REP cells, while little or no 4-1BB expression was found in the remaining live feeder cells (Figure A in Figure S1). However, no appreciable 4-1BBL was expressed in either sub-population (Figure B in Figure S1). Thus, CD8^+^ TIL induce 4-1BB expression after REP initiation, but little or no 4-1BBL is expressed by either the TIL or the feeders.

### Anti-4-1BB Antibody Increases CD8^+^ Percentage and Recovery during the REP

**Figure 1:**
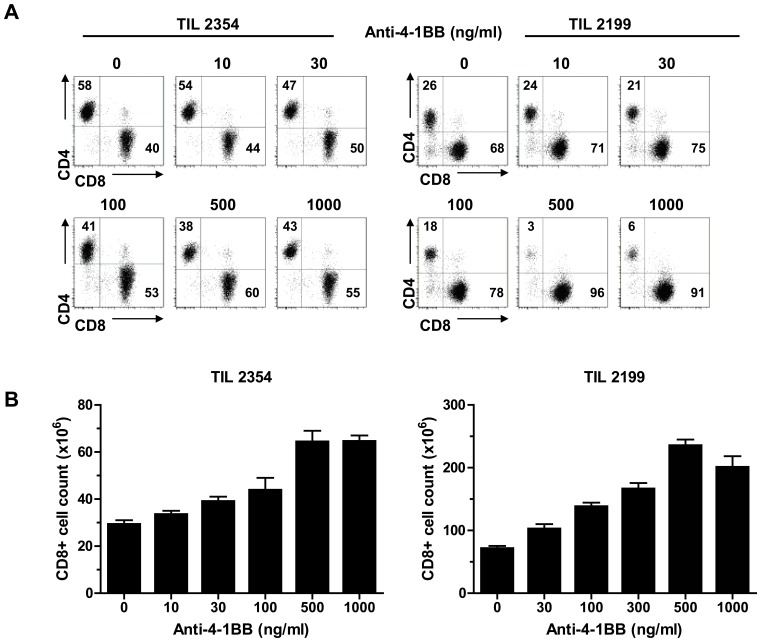
Addition of anti-4-1BB antibody to the REP increased in the percentage of CD8^+^ TIL. Anti-4-1BB antibody was added to the TIL on day 0 of the REP at the indicated concentrations. All other conditions were the same in each culture. After 14 days the cells were harvested and stained for CD4 and CD8 expression and viable cell counts were done using a hemocytometer following Trypan Blue staining. 4-1BB co-stimulation during the REP increased the frequency of CD8^+^ T cells in a dose-dependent fashion in a representative TIL lines #2354 and #2199 (**A**). The total yield of CD8^+^ T cells after the REP with different doses of anti-4-1BB is shown in two independent TIL lines (#2354 and #2199) (**B**). A dose-dependent increase in CD8^+^ T-cell yield was noted, with 500ng/ml of anti-4-1BB antibody being optimal. The results of triplicate cell counts ± standard deviation are shown.

The results above indicate that although CD8^+^ TIL up-regulate 4-1BB expression, there is no endogenous source of ligand activating 4-1BB co-stimulation. Thus, we tested the effects of an exogenous source of ligand by adding an agonistic anti-4-1BB mAb (BMS-663513) on the yield of CD8^+^ T cells during the REP. In the first set of experiments we tested the effects of different concentrations of anti-4-1BB added on day 0 (at the time of REP initiation). Addition of increasing concentrations of anti-4-1BB (0–1,000 ng/ml) resulted in an increasing frequency of CD8^+^ T cells with a decrease in the percentage of CD4^+^ T cells, as shown in the flow cytometry dot plots of two representative TIL lines (Figure 1A). This was manifested in an increased yield of CD8^+^ T cells at the end of the REP (Figure 1B) with a maximum frequency and yield of CD8^+^ T cells found at 500 ng/ml of mAb. Next, we determined the optimal day of addition of anti-4-1BB to maximize the frequency and yield of CD8^+^ T cells during the REP. Two TIL lines were tested by adding anti-4-1BB either on day 0, 1, 2, 3, or 5 of the REP. As shown in Figure S2, addition of the mAb on day 0 was optimal in both TIL lines tested. Thus, in all subsequent experiments anti-4-1BB was added at a dose of 500 ng/ml on day 0 of the REP. As a control, we also tested the effects of an agonistic anti-CD28 mAb added to the REP at this same dose in comparison to anti-4-1BB. CD28 is expressed on most pre-REP CD4^+^ and CD8^+^ TIL [8], [9]. However, as opposed to anti-4-1BB, addition of an agonistic anti-CD28 antibody did not increase the yield of CD8^+^ T cells (Figure S3).

**Figure 2:**
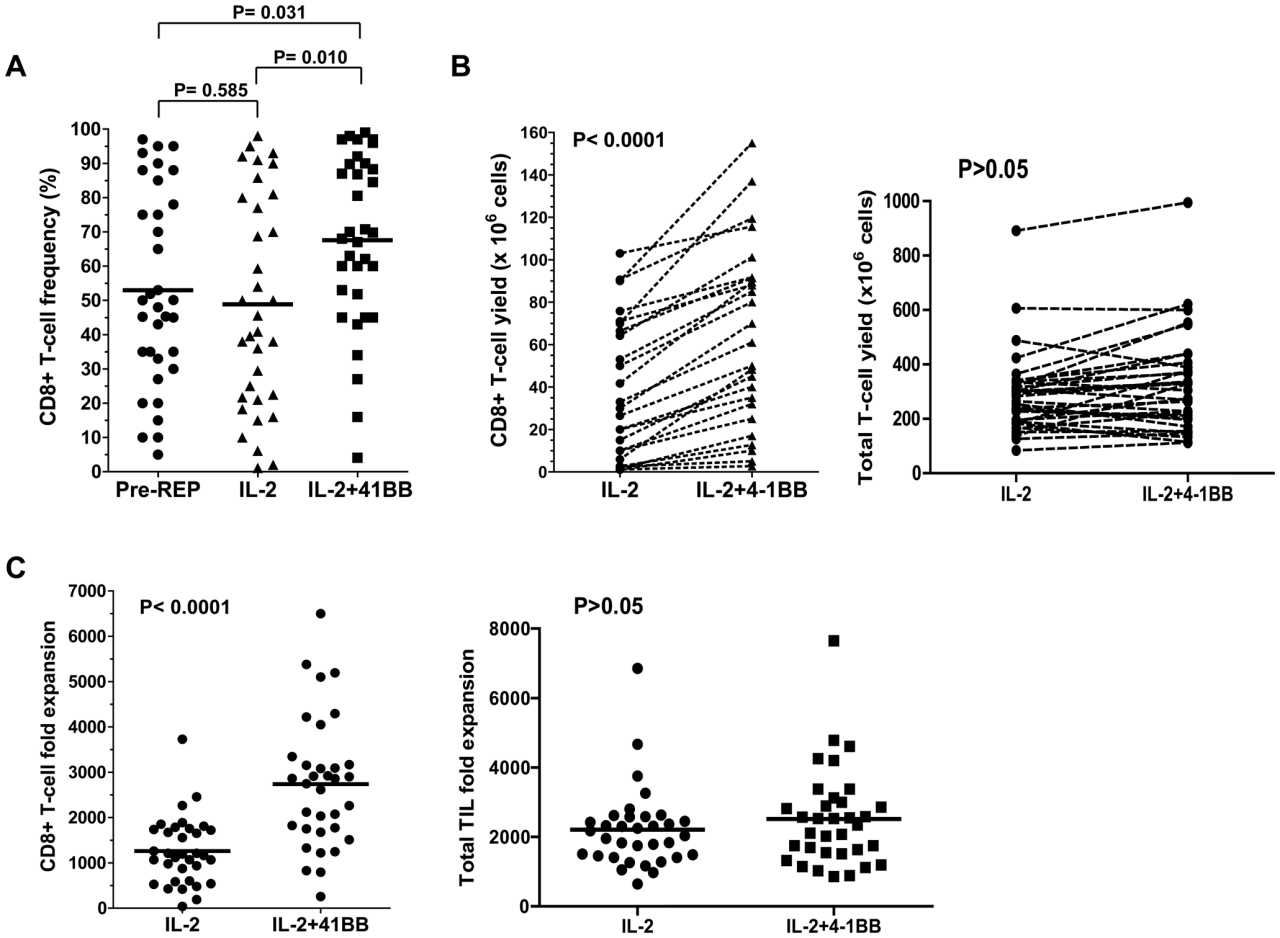
Addition of 4-1BB antibody in the REP increases CD8^+^ T-cell frequency and yield in a large cohort of patient samples (n = 34). The effects of addition of an optimal dose of anti-4-1BB (500 ng/ml), as determined previously, were tested in rapid expansions of TIL from 34 separate patient tumors. Addition of anti-4-1BB significantly increased the frequency of CD8^+^ T cells in the final TIL product in the patient population (**A**). CD8^+^ T-cell yield (**B**), and the fold expansion of CD8^+^ cells (**C**), was significantly increased when anti-4-1BB was added to the REP over the patient population. However, 4-1BB stimulation did not alter the total T-cell yield (**B**) or the total TIL fold expansion (**C**). Statistical analysis was done using the Wilcoxon signed rank test using biological relevance occurring when p<0.05.

In order to determine how reproducible the effects of 4-1BB co-stimulation were, we performed experiments with TIL from 34 different patients. An equal number of pre-REP TIL from each patient (0.13×10^6^ cells) were activated in a REP with anti-4-1BB added on day 0 (“4-1BB REP”) or without anti-4-1BB (“Control REP”) as control. As shown in Figure 2A, the anti-4-1BB REP significantly increased the frequency of CD8^+^ T cells recovered after the REP. Comparison of the yield and expansion of total CD8^+^ T cells for each TIL line also found a significant increase with the addition of anti-4-1BB (Figure 2B and 2C). In contrast the frequency and yield of CD4^+^ T cells exhibited an opposite trend (data not shown). The stimulation of the 4-1BB pathway, however, did not alter the total T-cell yield (Figure 2B right panel) or total TIL fold expansion (Figure 2C right panel). Thus, 4-1BB co-stimulation at the initiation of the REP reproducibly increases the frequency and yield of CD8^+^ T cells for adoptive cell therapy.

### 4-1BB Co-stimulation during the REP does not Restrict the TCR Vβ Repertoire

We wanted to address the possibility that although 4-1BB co-stimulation enhances the outgrowth of CD8^+^ T cells during the REP, it may lead to an oligoclonal expansion of certain CD8^+^ T-cell clones restricting the CD8^+^ T-cell repertoire after the REP. To test this, we sorted CD8^+^ T cells after rapid expansion with or without anti-4-1BB from 2 independent patient samples and performed Vβ spectratyping on isolated RNA. Analysis of the number of detected major Vβ subtypes and the number of CDR3 region lengths in each represented Vβ family revealed that, although some random gains and losses of Vβ CDR3 peaks occured in in either situation, the TCR repertoire of the 4-1BB-costimulated post-REP CD8^+^ T cells remained as diverse as in REPs that did not receive 4-1BB co-stimulation with no evident skewing towards any specific Vβ family evident (Figure S4).

### 4-1BB Co-stimulation during the REP Preserves CD28 Expression in CD8^+^ T cells

We have previously reported that many CD8^+^ TIL down-regulate CD28 expression after the REP and that these cells became hyporesponsive to re-stimulation with melanoma antigens such as MART-1 and were more susceptible to cell death [8]. The remaining CD8^+^CD28^+^ T cells had a superior survival and responsiveness to antigenic re-stimulation. Preservation of CD28 expression was also previously shown to be associated with longer telomere length and *in vivo* persistence of transferred TIL [8], [12]. CD27 is another effector-memory marker that can be down-modulated [9]. Studies at the National Cancer Institute (Bethesda, MD) have demonstrated that in melanoma patients receiving ACT, the total number of CD8^+^CD27^+^ TIL administered to patients was associated with improved clinical responses [4]. Thus, we went on to investigate the effects of the anti-4-1BB mAb in modulating both the extent of CD28 and CD27 expression.

**Figure 3:**
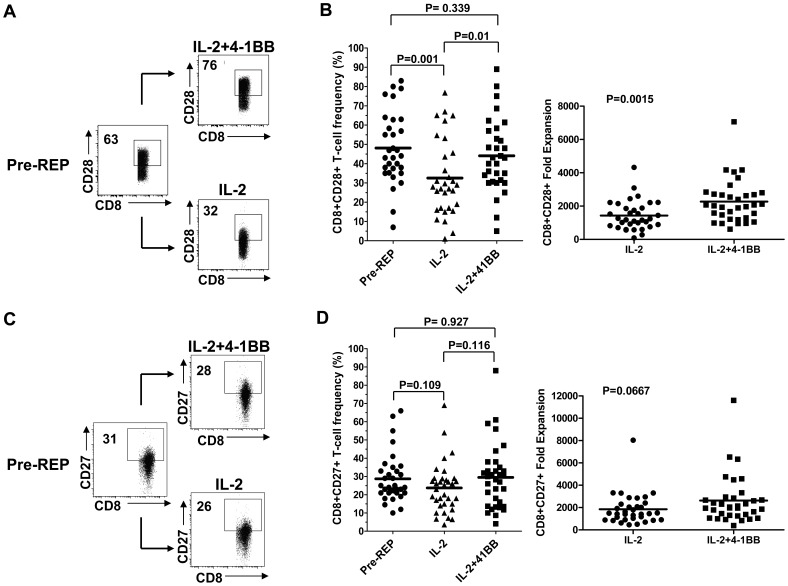
Addition of anti-4-1BB antibody maintained CD28 expression on CD8^+^ T cells during the REP in a large cohort of patient samples (n = 34). TIL from a representative patient was subjected to the REP with or without the addition of anti-4-1BB antibody and the cells stained for CD8 and CD28 (**A**) or CD8 and CD27 (**B**) and analyzed by flow cytometry both before the REP (pre-REP) and after the control (IL-2) REP or REP with anti-4-1BB (IL-2+4-1BB). Viable cells were gated and the frequency of CD28^+^ (A) or CD27^+^ (**C**) in the CD8^+^ subset analyzed. The change in CD28 or CD27 expression in the CD8^+^ T-cell subset during the REP with or without added anti-4-1BB was determined in 34 independent TIL lines (**B** and **D**). The left hand panels in B and D show the overall median difference in the frequency of CD28^+^ or CD27^+^ cells in the CD8^+^ subset over the entire patient population. The right hand panels in **B** and **D** show the fold expansions in the CD28^+^ or CD27^+^ populations. Statistical analysis was done using Wicoxon signed rank sum test using biological relevance occurring when p<0.05.

An example of the flow cytometry analysis of one TIL line is shown in Figure 3A and 3C. A significant loss of surface CD28 expression occured in the CD8^+^ TIL subset during the Control REP, while in the 4-1BB REP the frequency of CD28^+^ cells in the CD8^+^ subset remained stable (Figure 3A). In contrast, levels of CD27 expression did not decrease during the Control REP relative to pre-REP levels and anti-4-1BB did not appreciably alter CD27 expression (Figure 3C). In order to confirm these results, we analyzed the 34 separate patient TIL lines used previously for changes in CD28 and CD27 frequency and fold expansion in the CD8^+^ TIL subset (Figure 3B and D). Plotting the pre-REP, Control REP and 4-1BB REP found that the frequency of CD28^+^ TIL significantly decreased from an average of around 50% to 30% over the entire TIL sample set, a situation that was mostly reversed when anti-4-1BB was added to the REP (Figure 3B). The left hand graph in Figure 3B shows that this preservation of CD28 expression was not driven by a few lines that highly increased CD28 expression during the 4-1BB REP, but that in most individual TIL lines a loss of CD28 occurred that was regained with anti-4-1BB, as seen by the “V-shaped” pattern. As before, CD27 frequency levels and fold change over the 34 TIL lines tested was not significantly changed during either the Control or the 4-1BB REP from pre-REP levels in the CD8^+^ TIL subset (Figure 3D).

### Addition of 4-1BB Antibody to the REP Increases CD8^+^ Effector Phenotype

**Figure 4:**
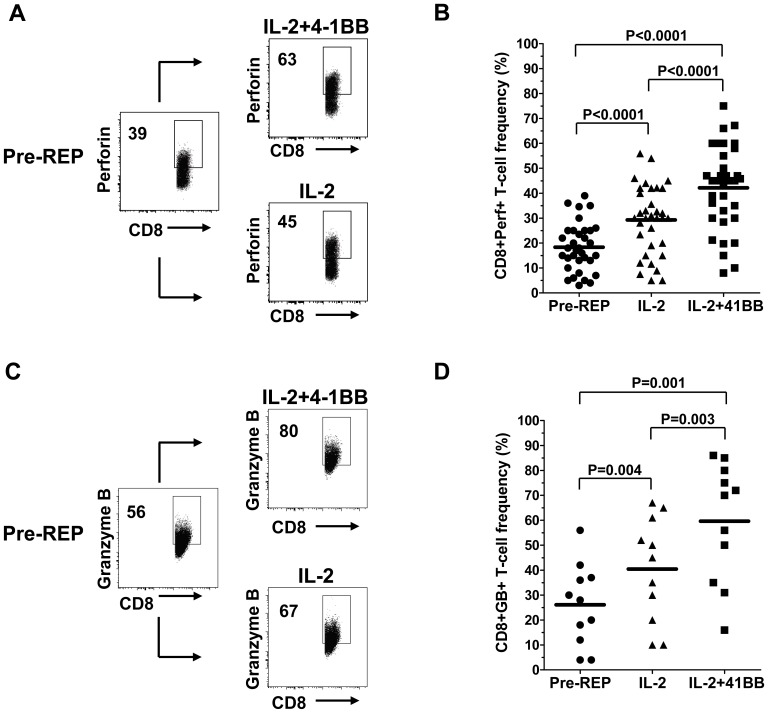
Addition of anti-4-1BB antibody increased Granzyme B (GB) and Perforin (Perf) expression in CD8^+^ T cells during the REP in multiple patient samples. TIL from a representative patient was subjected to the REP with or without the addition of anti-4-1BB antibody and the cells stained for CD8 and Perf (**A**) or CD8 and GB (**B**) and analyzed by flow cytometry both before the REP (pre-REP) and after the control (IL-2) REP or REP with anti-4-1BB (IL-2+4-1BB). Viable cells were gated and the frequency of Perf^+^ (**A**) or GB^+^ (**C**) in the CD8^+^ subset analyzed. The change in Perf or GB expression in the CD8^+^ T-cell subset during the REP with or without added anti-4-1BB was determined in larger cohort of patients TIL samples (**B** and **D**). 4-1BB co-stimulation during the REP significantly increased Perf expression in CD8^+^ T cells in 34 separate TIL line tested (**B**) and increased GB expression in 11 separate TIL lines tested (**D**). Statistical analysis was done using Wicoxon signed rank sum test using biological relevance occurring when p<0.05.

A number of hallmarks are used to differentiate CD8^+^ T cells that gain effective cytotoxic T lymphocyte (CTL) or killing function. These include the gain in intracellular expression of cytolytic granule molecules, such as perforin (Perf) and granzyme B (GB). Increased expression of the T-box transcription factor eomesodermin (Eomes), helping drive Perf expression, can also be seen [18]. In some cases, later stage or more highly differentiated effector CD8^+^ T cells gain expression of an NK marker called killer cell lectin like receptor subfamily G member 1 (KLRG-1), a marker usually associated with senescent, end-stage CD8^+^ T cells with low proliferative capacity [18], [19]. Using flow cytometry analysis as before, we analyzed changes in Perf, GB, Eomes, and KLRG-1 expression in the CD8^+^ TIL subset. Perf changes were monitored in the 34 separate patient TIL lines, while GB was determined in a subset of 17 of these lines (Figure 4B and D). While both Perf and GB frequencies increased during the REP, addition of anti-4-1BB induced significantly higher frequency of Perf and GB CD8^+^ T cells (Figure 4A and C). Eomesodermin and KLRG-1 expression changes were studied in 10 different TIL lines. No significant differences however were found between the Control REP and 4-1BB REP in each case, although Eomes did show a tendency to increase at variable levels in many of the TIL lines (Figure S5).

### Analysis of Cytokine Secretion and Anti-tumor CTL Activity

**Figure 5:**
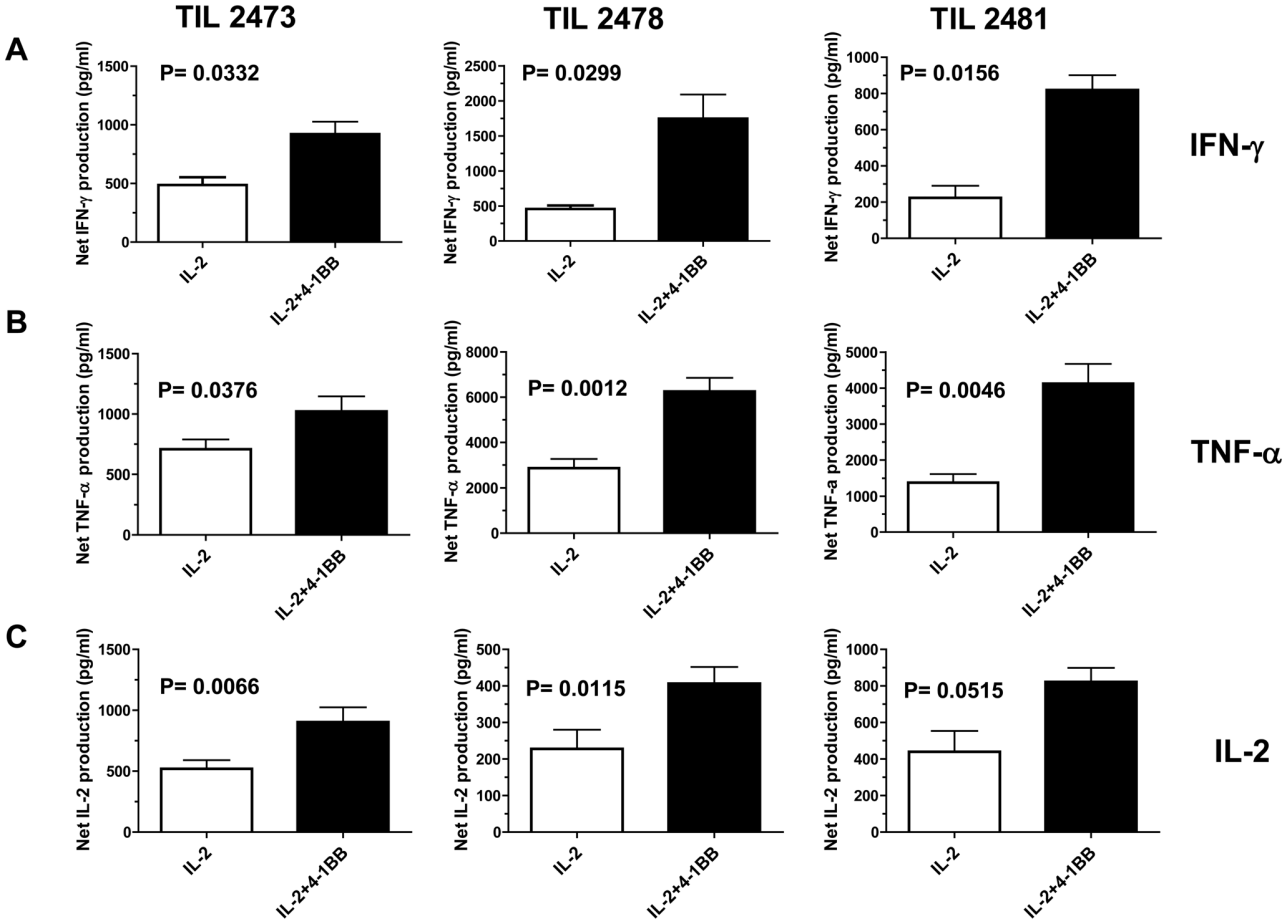
TIL isolated after rapid expansion with anti-4-1BB antibody displayed an increased ability to secrete IFN-γ, TNF-α, and IL-2 after TCR re-stimulation. Melanoma TIL rapidly expanded with or without the addition of the anti-4-1BB antibody were re-stimulated with anti-CD3 antibody in 96-well plates. Culture supernatants were collected after 24 hours and assayed using anti-cytokine beads for IFN-γ, TNF-α, and IL-2 using a Luminex-100 system. Results from 3 different TIL lines comparing the control (IL-2) group with the IL-2+4-1BB group are shown for IFN-γ (**A**) TNF-α (**B**), and IL-2 (**C**). In each case the net production of the cytokines was calculated by subtracting the control wells (no anti-CD3) from the wells that had anti-CD3. The averages and standard deviation of triplicate wells are shown in each case. A paired student’s t-test was used to determine statistical significance between groups with p<0.05 indicating statistical significance.

**Figure 6:**
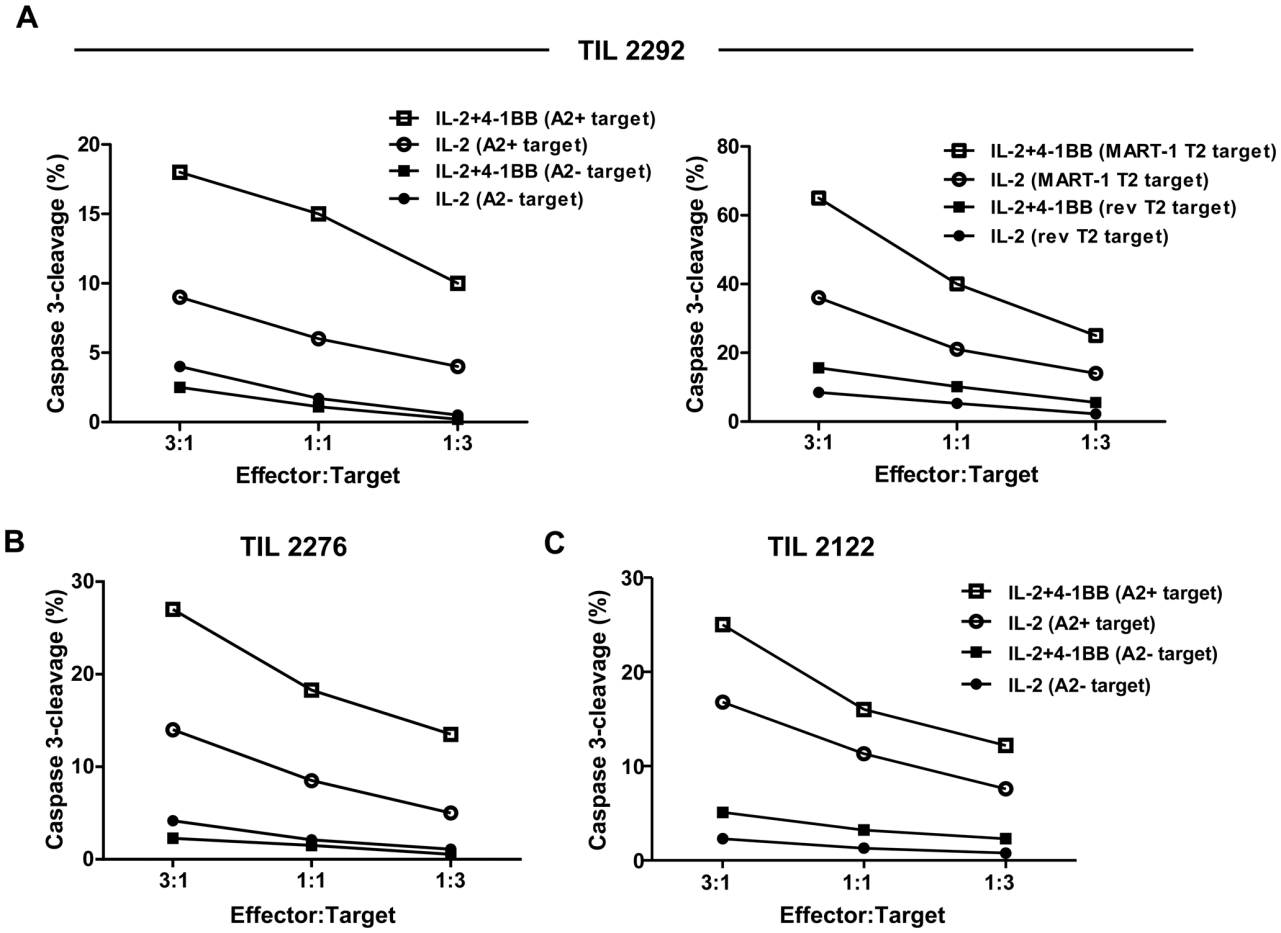
Addition of anti-4-1BB antibody to the REP led to increased post-REP TIL tumor antigen-specific CTL activity. Melanoma TIL from HLA-A0201^+^ patients with a significant population of CD8^+^MART-1 tetramer^+^ cells were rapidly expanded with or without anti-4-1BB as before. The post-REP TIL were sorted by FACS for CD8^+^ T cells and assayed for tumor antigen-specific CTL activity using a flow-cytometry-based assay that measures caspase-3 cleavage in target cells. The results of three different patient TIL lines are shown. The top panels (**A**) show the CTL activity of TIL #2292 using the melanoma cell line 624 (HLA-A0201^+^) and the control HLA-A-unmatched line 938 as targets (left side), or MART-1 peptide-pulsed T2 target cells as targets (right side). The bottom panels (**B**) show the CTL activity of two other HLA-A0201^+^ TIL lines (#2276 and #2122) against 624 or 938 cells with similar results. In all cases (**A** and **B**) the levels of non-specific killing were markedly lower.

The results above suggested that enhancement of 4-1BB co-stimulation early during the TIL REP generates CD8^+^ T cells with increased effector function. We determined the effector activity of the TIL by testing their ability to produce cytokines in response to TCR stimulation post-REP and their ability to kill tumor cells or targets pulsed with melanoma antigen peptides. As before, TIL from different patient pre-REP lines were rapidly expanded with or without added anti-4-1BB antibody. The post-REP TIL were then sorted by FACS to isolate the CD8^+^ subset, washed, and rested for 5–6 h, and then assayed for cytokine production or CTL activity. As shown in Figure 5, sorted CD8^+^ T cells from REP cultures including anti-4-1BB secreted significantly higher amounts of IFN-γ, TNF-α, and IL-2 following CD3 activation. CTL activity was determined using a previoulsy published assay measuring capsase 3 cleavage in target cells by flow cytometry [16]. Figure 6 shows the results of post-REP CTL assays on three different HLA-A0201^+^ patient TIL lines using an HLA-A0201-matched melanoma cell line target (cell line 624) and an HLA-A-unmatched melanoma target (cell line 938), or T2 target cells pulsed with a an HLA-A0201 MART-1 peptide epitope. We found that the sorted CD8^+^ T cells from the anti-4-1BB-treated REP cultures had higher levels of specific killing activity than cells from control REP cultures (Figure 6). Levels of non-specific killing were low in all cases (Figure 6). Staining with an HLA-A0201 MART-1 peptide HLA tetramer found that the frequency of MART-1-specific cells in was only slightly higher in the post-REP TIL from the 4-1BB costimulate expansions (Figure S6) suggesting that the enhanced killing activity (at least in the case of MART-1 antigen) is not due to a higher frequency of melanoma antigen-specific CD8^+^ T cells after the REP with anti-4-1BB.

### Increased bcl-2 Expression and Post-REP Cell Survival in TIL Rapidly Expanded with 4-1BB Co-stimulation

**Figure 7:**
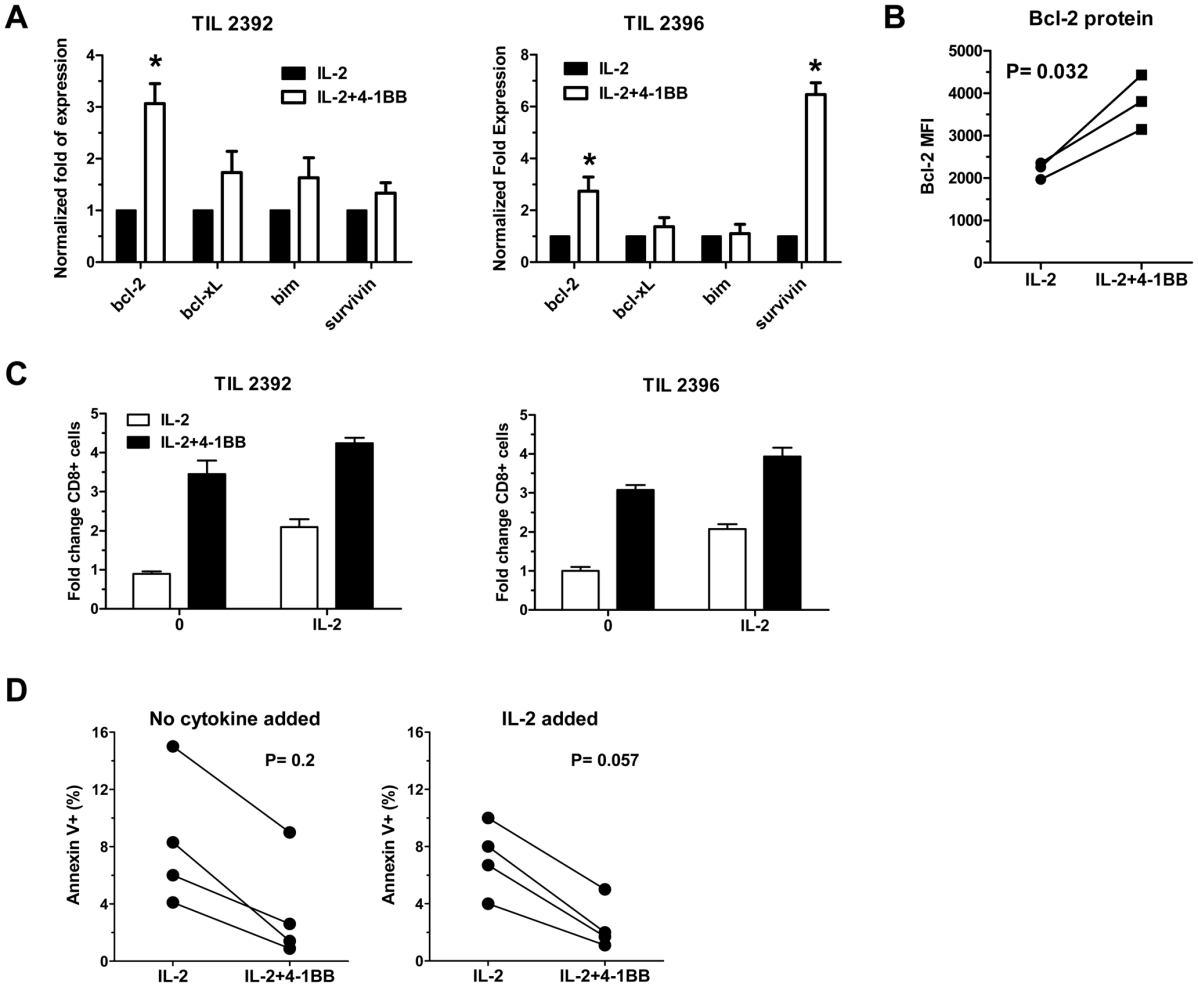
Increased expression of anti-apoptotic molecules and improved survival in post-REP TIL that received 4-1BB co-stimulation during the REP. RNA from post-REP TIL that were rapidly expanded with or without anti-4-1BB were subjected to real-time PCR (qRT-PCR) analysis for bcl-2, bcl-xL, bim, and survivin (**A**). The results of two representative patient TIL lines are shown (#2392 and #2396). Each PCR reaction was ran in triplicate with a CV of <5% for all assays. The results with the IL-2+4-1BB REP were normalized against the levels of gene expression in the control (IL-2) REP, as indicated with the 1-fold expression for the control REP for each gene. The increase in bcl-2 expression in the IL-2+4-1BB group was confirmed in three different TIL samples by intracellular staining for bcl-2 using flow cytometry (**B**). The post-REP TIL were also analyzed for their ability to survive and further expand when re-cultured with or without IL-2 (100 U/ml) for 5 days, as described in the Materials and Methods (**C** and **D**). Viable cell counts together with flow cytometry analysis for CD8 expression was performed to calculate the fold change in CD8^+^ T cells after the re-culturing period (**C**). TIL from 4-1BB REP (IL-2+4-1BB) or control REP (IL-2) cultures from 4 different patients were also subjected to staining with Annexin V and 7-AAD after re-plating without or with 200 IU/ml IL-2 for 5 days and the frequency of Annexin V^+^7-AAD^−^ (apoptotic cells) were determined (**D**).

4-1BB has been shown to prevent apoptosis by up-regulating anti-apoptotic molecules, such as bcl-xL and bcl-2 [17]. We have previously shown that 4-1BB protects post-REP TIL from activation-induced cell death [14]. Therefore, we wanted to determine whether the addition of anti-4-1BB during the REP resulted in any change in expression of the major bcl-2 family members that are anti-apoptotic (bcl-2 and bcl-xL) or pro-apoptotic (bim). We also looked at the levels of survivin, a member of the inhibitor of apoptosis family that has also been shown to be induced by TNF-R family signaling, such as OX40 [20]. Post-REP TIL from two patients were isolated as before and subjected to real-time quantitiative PCR analysis. Interestingly, bcl-2 and not bcl-xL was consistently up-regulated in TIL that received 4-1BB co-stimulation during the REP, while bim was not altered significantly (Figure 7A). We also found a significantly higher expression of the Survivin gene in the 4-1BB costimulated TIL, although this was more nominal than with bcl-2 (Figure 7A). We also confirmed the increased expression of bcl-2 using flow cytometry. We found an increased expression in the bcl-2 mean fluorescence intensity (MFI) in TIL isolated from the 4-1BB REP compared to the control (IL-2) REP (Figure 7B). We also analyzed the cell survival ability of post-REP TIL when re-cultured without cytokine or with added IL-2 for 5 days by determining the recovery of viable CD8^+^ cells and their level of apoptosis. IL-2 therapy is given immediately after TIL adoptive transfer into patients and, thus, we were interested in how the cells respond to IL-2 following the REP. The cells were harvested and washed three times to remove any remaining cytokine in the REP and replated. Remarkably, CD8^+^ TIL from both patients that received 4-1BB co-stimulation during the REP exhibited a 3–4- fold increase in cell number with or without added IL-2 (200 IU/ml), while control REP CD8^+^ TIL only further expanded with additional IL-2 and had a reduction in the number of cells when no exogenous IL-2 was provided (Figure 7C). This improved yield of TIL from 4-1BB REP cultures when post-REP cells were re-plated with or without added IL-2 was also reflected in a lower percentage of apoptotic (Annexin V^+^) cells (Figure 7D). Although the data from the four separate TIL lines were not statistically significant due to the different intrinsic apoptosis sensitivities of each line, there was a clear trend towards a decrease in Annexin V^+^ cells in each case with TIL from the 4-1BB REP cultures (IL-2+4-1BB) versus control REP cultures (IL-2) when the post-REP cells were re-plated without IL-2 or with added IL-2 (Figure 7D).

### 4-1BB Co-stimulation during the REP Enhances Responsiveness of MART-1 Specific TIL to Antigen Re-stimulation

**Figure 8:**
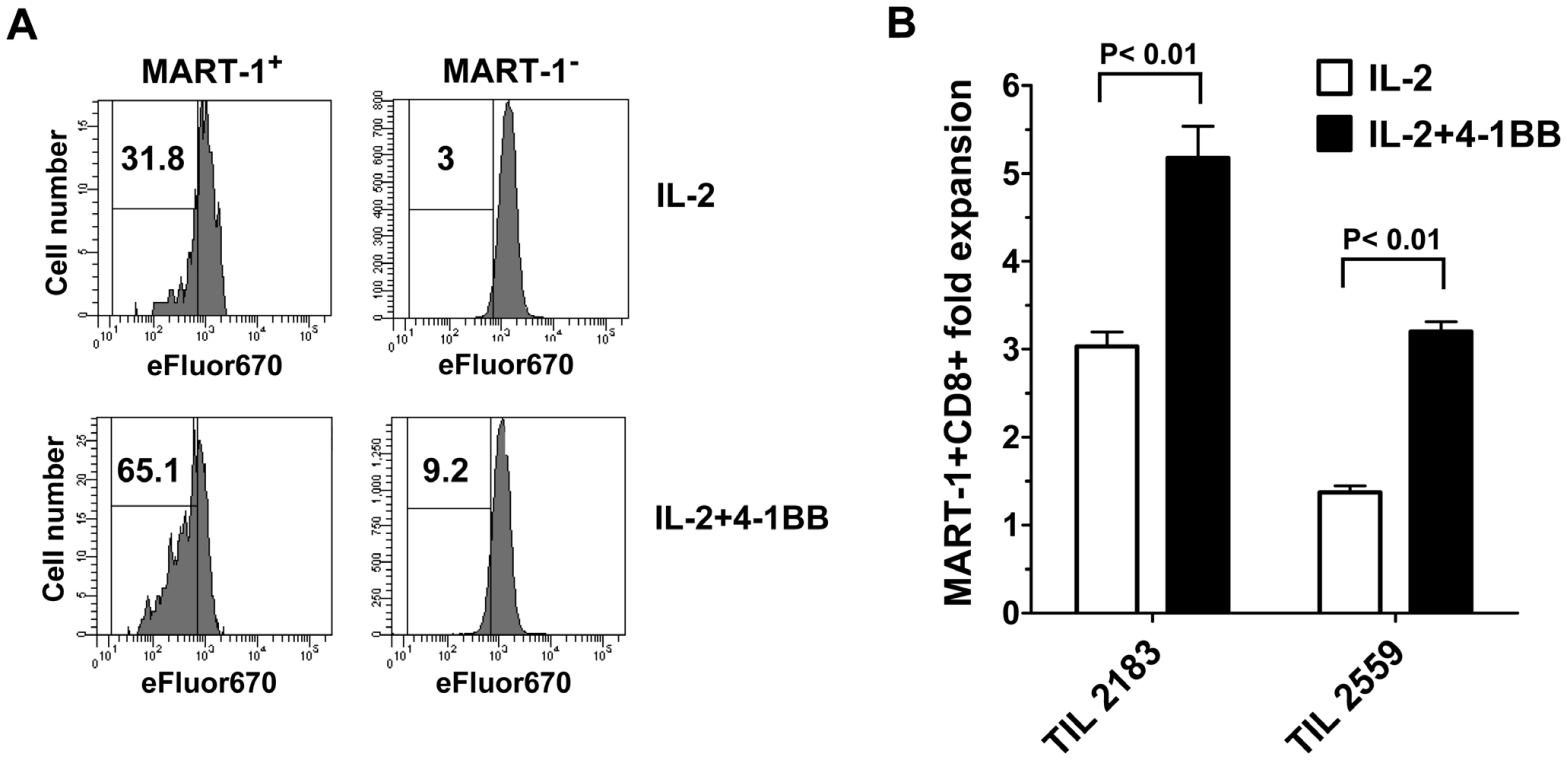
CD8^+^ MART-1-reactive TIL provided 4-1BB co-stimulation during the REP have a greater proliferative response to re-stimulation with MART-1 peptide. TIL from HLA-A0201^+^ patients were rapidly expanded with or without added anti-4-1BB antibody. The cells were labeled with eFluor670 dye and re-stimulated with HLA-A0201^+^ MART-1 peptide-pulsed DC, as previously described [8]. The post-REP TIL were labeled with the cell division dye eFluor670 (Invitrogen) and re-stimulated with HLA-A0201-matched mature DC pulsed with MART-1 peptide for 7 days. The cells were harvested and stained for CD8 and MART-1 tetramer and analyzed by flow cytometry for the frequency of divided (eFluor670^low^) in the gated CD8^+^ MART-1 tetramer^+^ subset. One representative experiment out of two is shown. As shown in **A**, CD8^+^ TIL isolated from 4-1BB costimulated REP cultures (#2183) had an enhanced response to MART-1 peptide re-stimulation, as shown by the increased number of cell divisions measured by eFluor670 dilution in the CD8^+^ MART-1 tetramer^+^ gated cells. TIL isolated from REP cultures that received anti-4-1BB also exhibited an increase in the fold expansion of CD8^+^ MART-1 tetramer^+^ cells following re-stimulation of the post-REP TIL with MART-1 peptide pulsed DC as shown in experiments with two separate TIL lines (#2183 and #2559) (**B**).

Previously, we reported that MART-1-specific CD8^+^ T cells that lose CD28 become hypo-responsive to antigenic re-stimulation by peptide-pulsed DC following the REP. We were therefore interested in whether additional 4-1BB co-stimulation during the REP yields more CD8^+^ T cells that may be more responsive to antigenic re-stimulation following the REP. CD8^+^ TIL from HLA-A0201^+^ patients that had a significant population of MART-1 peptide tetramer^+^ CD8^+^ T cells were rapidly expanded with or without 4-1BB co-stimulation. Similar starting numbers of CD8^+^ MART-1 tetramer^+^ TIL were used (data not shown). The cells were labeled with eFluor670 dye and re-stimulated with HLA-A0201^+^ MART-1 peptide-pulsed DC, as previously described [8] and cell counts were done using trypan blue and a hemocytometer. As shown in Figure 8A, CD8^+^ TIL isolated from 4-1BB co-stimulated REP cultures had an enhanced response to MART-1 peptide re-stimulation, as shown by the increased number of cell divisions measured by eFluor670 dilution in the CD8^+^ MART-1 tetramer^+^ gated cells (Figure 8A). Figure 8B shows the results of two experiments with HLA-A0201^+^ post-REP TIL re-stimulated with HLA-A0201^+^ MART-1 peptide-pulsed DC. In both cases, the fold increase in gated CD8^+^MART-1 tetramer^+^ cells was significantly higher in the samples that originally received anti-4-1BB in the REP (IL-2+4-1BB).

## Discussion

Adoptive transfer of *ex vivo* expanded autologous TIL has emerged as a powerful therapy for unresectable stage III and stage IV metastatic melanoma in multiple Phase II clinical trials [1], [3], [4]. We have found that both the total number of TIL and CD8^+^ T cells within the infused TIL are critical in mediating tumor regression associated with improved overall survival in melanoma patients receiving TIL therapy [3]. Moreover, specific subsets of effector-memory cells within the CD8^+^ subset are emerging to have enhanced persistence and anti-tumor properties [3], [9], [12], [21]. The key to successful adoptive cell therapy with TIL is the generation of T cells with memory properties that can survive and persist *in vivo* long enough, as well as optimal effector properties, that can in turn mediate tumor regression both in the short-term and long-term after TIL infusion. Co-stimulatory signaling during TCR stimulation is critical in generating these long-lived effector-memory cells [21]–[24]. However, little is known about the role of specific co-stimulatory signaling molecules in regulating the outcome of melanoma TIL expansion and the quality of the T cells, especially CD8^+^ T cells for adoptive transfer. The REP is a key step generating the large numbers of cells (in the billions) used for adoptive cell therapy. However, we and others have found that it actually generates a sub-optimal population of differentiated effector-memory cells that have not only variable effector (tumor cell killing) properties, but that can also be hypo-responsive to re-stimulation by melanoma antigens and susceptible to activation-induced cell death (AICD); these have been associated with a loss of CD28, and to lesser extent CD27, and memory function [8]. We had previously shown that post-REP CD8^+^ TIL that have lost CD28 retained the capacity to up-regulate 4-1BB and could be protected from AICD by provision of 4-1BB co-stimulation [14]. Here, we were interested in what the effects of 4-1BB co-stimulation earlier in the process (at the start of the REP) were on final TIL phenotype and function.

Currently, the TIL REP is performed using an excess of irradiated allogeneic or autologous feeder cells [13]. Despite many years of this REP method being performed very little is known about the exact functional properties the feeders have in facilitating TIL expansion. It has been assumed that they provide a source of Fc receptors to crosslink the anti-CD3 antibody used to activate TIL as well as co-stimulatory signals to help drive T-cell expansion. However, as we have shown here, the feeder cells were found to express very little, if at all, 4-1BB ligand (4-1BBL), a critical co-stimulatory molecule for CD8^+^ T cells [24], [25]. Although we have not measured the expression of CD28 ligands, CD80 and CD86, as well as ligands for negative co-stimulatory molecules (e.g., PD-1), this should also be done in future experiments. We also found that the TIL themselves (as expected) did not express 4-1BBL. Thus, a lack of adequate 4-1BB co-stimulation during the REP could account, at least in part, for the loss of memory-effector function of the CD8^+^ T cells during the REP and why many times CD4^+^ T cells can outgrow the CD8^+^ T cells. The improved expansion of CD8^+^ T cells, together with the improved preservation of CD28 expression, antigen recall responses, and enhanced anti-tumor effector functions when an agonistic anti-4-1BB was added early in the REP process indicate that a lack of adequate 4-1BB co-stimulation in the current TIL REP protocols is indeed a critical issue. This suggests that addition of anti-4-1BB should be considered as part of the routine clinical REP procedure, and with the availability of clinical grade (GMP-grade) agonistic monoclonal antibodies (e.g., BMS 663513) [26] this should not be a problem. Another approach could be the use of so-called “artificial antigen-presenting cells” (aAPC) based on the K562 cell line that has been engineered to express both CD86 and 4-1BBL in addition to CD64 (FcγRI) for presenting anti-CD3 to the T cells [25], [27], [28]. Ye et al. demonstrated that utilizing a K562-based aAPC that expressed the CD137L (4-1BBL) compared to aAPC that did not express the CD137L increased TIL expansion and yielded a greater CD8^+^ TIL frequency after expansion [29]. We are currently testing a first generation K562 aAPC expressing CD64, CD86, and 4-1BBL for its ability to expand melanoma TIL and whether it enhances the expansion of CD8^+^ T cells with similar properties as those found here. However, the specific role of the 4-1BB pathway using these aAPC will need to be explored by blocking 4-1BBL contact with the T cells. Another question regarding the aAPC technology is the relative strength of providing 4-1BB co-stimulation to the TIL using the ligand versus an agonistic antibody. It is possible that an agonistic antibody may be optimal. Moreover, the strength of 4-1BB co-stimulation may be better controlled by using different concentrations of antibody as compared to the ligand expressed on the aAPCs.

Another aspect of our work was to look at how provision of 4-1BB co-stimulation during the TIL REP affected the survival and proliferation potential of post-REP CD8^+^ T cells that would be part of the infusion product, especially in response to re-exposure to IL-2. IL-2 therapy (both high-dose and low-dose regimens) is given immediately after TIL infusion [2], [3]. Currently, although lymphodepletion given to patients prior to TIL adoptive transfer helps remove cytokine sinks and regulatory T cells (Tregs), many of the transferred T cells still do not persist despite exogenous IL-2 treatment [30], [31]. In this context, it is highly relevant that the post-REP CD8^+^ TIL that were previously provided 4-1BB co-stimulation exhibited enhanced survival properties, as determined by increased anti-apoptotic gene expression and their enhanced survival and continuous expansion, when re-cultured for a number of days with or without added IL-2. In addition, in a melanoma antigen re-stimulation assay using MART-1 peptide-pulsed DCs, we found that post-REP CD8^+^ MART-1 tetramer^+^ TIL that received previous 4-1BB co-stimulation exhibited a superior proliferative response. These results suggest that TIL provided with 4-1BB co-stimulation during the REP may have improved persistence and expansion *in vivo* especially early on after infusion during IL-2 therapy and after contact with melanoma antigens *in vivo*. In this regard, it is noteworthy that 4-1BB co-stimulation increased CD28 expression and critical effector molecules needed for tumor killing (Perforin, GB, Eomes) without significantly increasing KLRG-1, a marker for end-stage effector cells approaching senescence [20], [32]. IL-15 has also been shown to induce 4-1BB on CD44^high^ CD8^+^ memory cells [33]. Thus, it is possible that addition of IL-15 to the TIL REP together with anti-4-1BB antibodies could further promote the maintenance of CD28 expression, although we did not determine this in the study here. Another possible way to further increase the yield of CD8^+^CD28^+^ memory cells in the REP is combine 4-1BB costimulation with a combination of IL-15 and IL-21 as substitute growth-promoting cytokines. We have previously found that this cytokine combination increases the yield of CD8^+^CD28^+^ T cells post-REP [8]. It would be interesting therefore to combine IL-15 and IL-21 together with anti-41BB to see if CD28 expression is increased even further.

Our data raises the prospect that provision of antibodies to other TNF-R co-stimulatory molecules, such as OX40, CD27, HVEM, alone or in different combinations with or without anti-4-1BB, may yield even further enhanced TIL products for adoptive transfer with even further enhanced effector-memory properties. Addition of the anti-4-1BB may also synergize with enhanced costimulation through CD28 in the REP using anti-CD28 antibodies that may lead to a more persistent cell surface expression of 4-1BB [34]. In initial experiments, we tested an agonistic anti-OX40 antibody (provided from Dr. Andrew Weinberg, Providence Portland Medical Center, Portland, OR) in the REP to expand TIL but found that anti-OX40 facilitated CD4^+^ TIL expansion rather than CD8^+^ TIL expansion. This was expected, as OX40 is mainly up-regulated on activated CD4^+^ T cells [17]. Although our study here primarily focused on the role of CD8^+^ TIL in ACT, CD4^+^ T cells present in adoptively-transferred TIL can also play a role in mediating a response in ACT. Studies have shown a positive role for CD4^+^ T cells in survival and persistence of memory CD8^+^ T cells in mouse models [35], at present the role of co-infused CD4^+^ TIL and CD4^+^ ‘T-cell help’ for CD8^+^ TIL has not been investigated. We have found that clinical response to TIL therapy was strongly associated with a higher frequency of CD8^+^ T cells infused in the expanded TIL product [3]. However, in all responding patients, at least some CD4^+^ T cells were still infused and whether these CD4^+^ cells were critical is unclear at present. We are currently addressing this question by longitudinally tracking the persistence and expansion or contraction of original CD4^+^ versus CD8^+^ T-cell clones from adoptively-transferred TIL in the blood from treated patients to determine whether there is a correlation with improved CD8^+^ TIL persistence with a critical threshold frequency of CD4^+^ T cells in the original infused TIL.

The provision of antibodies to other co-stimulatory molecules, may in fact eliminate the need for PBMC feeder cells altogether and allow for a more practical approach to the TIL REP using either antibody-coated culture vessels or soluble antibodies, or using nanoparticles linked to either the actual ligands for these receptors or aptamers binding these receptors. In this regard, it will also be important to determine whether the added anti-4-1BB antibody used here was bound by feeder cells expressing Fc receptors and presented to the activated TIL or whether it was active in a soluble fashion. Our results with delayed addition of anti-4-1BB however suggests the former because of the loss of activity after day 1 of the REP after which the irradiated feeder cells die.

In summary, we have demonstrated that utilizing an agonistic anti-4-1BB antibody to augment the TNF-R family member 4-1BB pathway during melanoma TIL expansion significantly improves the phenotype and function of tumor-reactive CD8^+^ CTL. We believe this approach could greatly improve TIL persistence and anti-tumor activity *in vivo* after adoptive transfer into patients.

## Supporting Information

Figure S1 **4-1BB is expressed on CD8+ TIL within the first 2 days of REP initiation.** Pre-REP TIL were stained for the expression of CD8, 4-1BB, and 4-1BBL, as shown. The REP was then set up with the TIL being labeled with CFSE prior to being added to the flask for the TIL expansion in order to be able to distinguish the TIL from excess of irradiated PBMC feeder cells added. On day 1 and day 2 of the REP, the cells were harvested from the flasks and stained for the expression of CD3, CD8, 4-1BB and 4-1BBL. For analysis of the TIL, the CFSE^+^ viable cells were gated, for the feeders, the CFSE- viable feeders were analyzed. We found that the TIL up-regulated 4-1BB on the CD8^+^ subset, while the PBMC feeder cells had much less 4-1BB expression (**A**). In contrast, both the CD8^+^ TIL and the PBMC feeders expressed only low levels of 4-1BBL (**B**). No 4-1BBL expression was detected on the CD4^+^ TIL on day 1 or day 2, or in the pre-REP cells (data not shown).
(TIF)

Figure S2 **The optimal day to add the anti-4-1BB antibody was day 0 of the REP for CD8^+^ TIL expansion.** The TIL were subjected to the REP with or without 500 ng/ml of the anti-4-1BB antibody added on different days of the REP (Day 0, 1, 2, 3, or 5), as indicated. On day 14 of the REP, the post-REP TIL were analyzed for the expression of CD8 on the viable population by flow cytometry. The highest increase in CD8^+^ T-cell frequency was observed when anti-4-1BB antibody was added on day 0 of the REP (**A**). Addition of anti-4-1BB on Day 0 also resulted in the highest change in the total yield of CD8^+^ T cells after the REP (**B**). The results shown are the average of triplicate cell counts after the REP ± standard deviation. A two-way ANOVA found that the Day 0 CD8^+^ T-cell count was significantly higher (p<0.05) than in the pre-REP TIL as well as for all other time points of anti-4-1BB addition (**B**).
(TIF)

Figure S3 **Comparison of the addition of agonistic anti-4-1BB and agonistic anti-CD28 to the TIL REP.** Melanoma TIL from 2 patients were subjected to the REP with or without addition of anti-4-1BB (500 ng/ml) or anti-CD28 (500 ng/ml) added during the REP initiation. Post-REP TIL were harvested, counted, and stained for the expression of CD8, CD27, and CD28. Gating was done on the viable cells. Addition of anti-4-1BB antibody increased the yield of CD8^+^ T cells over the control (IL-2) REP significantly more than addition of anti-CD28. An average of 3 independent cell counts are shown with bars indicating standard deviation. Statistical analysis was done using a two-way ANOVA with Bonferroni post-tests. An asterisk above the bar indicates a p-value of <0.05 relative to the control (IL-2) REP. In each case anti-4-1BB induced a significant increase in CD8^+^ T-cell yield over anti-CD28.
(TIF)

Figure S4 **TCR Vβ repertoire is not restricted in the post-REP TIL that received 4-1BB co-stimulation**. RNA was isolated from pre-REP TIL. These TIL then underwent the REP with or without the addition of the anti-4-1BB antibody. RNA was isolated on the post-REP TIL and Vβ spectratyping analysis was done on pre-REP and the post-REP TIL. In 2 representative TIL lines 2549 and 2550, we found that the TIL isolated from the IL-2 or IL-2+4-1BB REP retained a diverse TCR Vβ repertoire without any increased oligloclonality.
(TIF)

Figure S5 **Increased expression of EOMES in TIL isolated after the REP with anti-4-1BB antibody, with no significant change of KLRG-1 expression.** The TIL subjected to the REP with or without the anti-4-1BB antibody were stained for CD8 and the expression of T-box transcription factor Eomesodermin (EOMES) (**A**) and Killer cell lectin like receptor subfamily G member 1 (KLRG1) (**B**). 4-1BB co-stimulation during the REP led to an increase in EOMES^+^ (**A**) in the CD8^+^ population (n = 21). However, there was no difference in expression of KLRG-1 (**B**) in the CD8^+^ population (n = 11). Statistical analysis was done using the Wilcoxon signed rank test with biological relevance occurring when p<0.05.
(TIF)

Figure S6 **4-1BB stimulation does not increase the frequency of MART-1-specific cells.** TIL were expanded with or without the anti-4-1BB antibody. Post-REP TIL were stained for CD8 and MART-1 tetramer. FACS The TIL were gated on the live population and analysis of the both types of post-REP TIL found that the percentage of CD8^+^ MART-1-specific cells was similar in 3 representative TIL lines
(TIF)
