# Supplementary figures and images for: Co-Stimulation through 4-1BB/CD137 Improves the Expansion and Function of CD8+ Melanoma Tumor-Infiltrating Lymphocytes for Adoptive T-Cell Therapy
Source: PLoS One. 2013 Apr 1;8(4):e60031. doi: 10.1371/journal.pone.0060031 (PMC3613355; doi:10.1371/journal.pone.0060031)

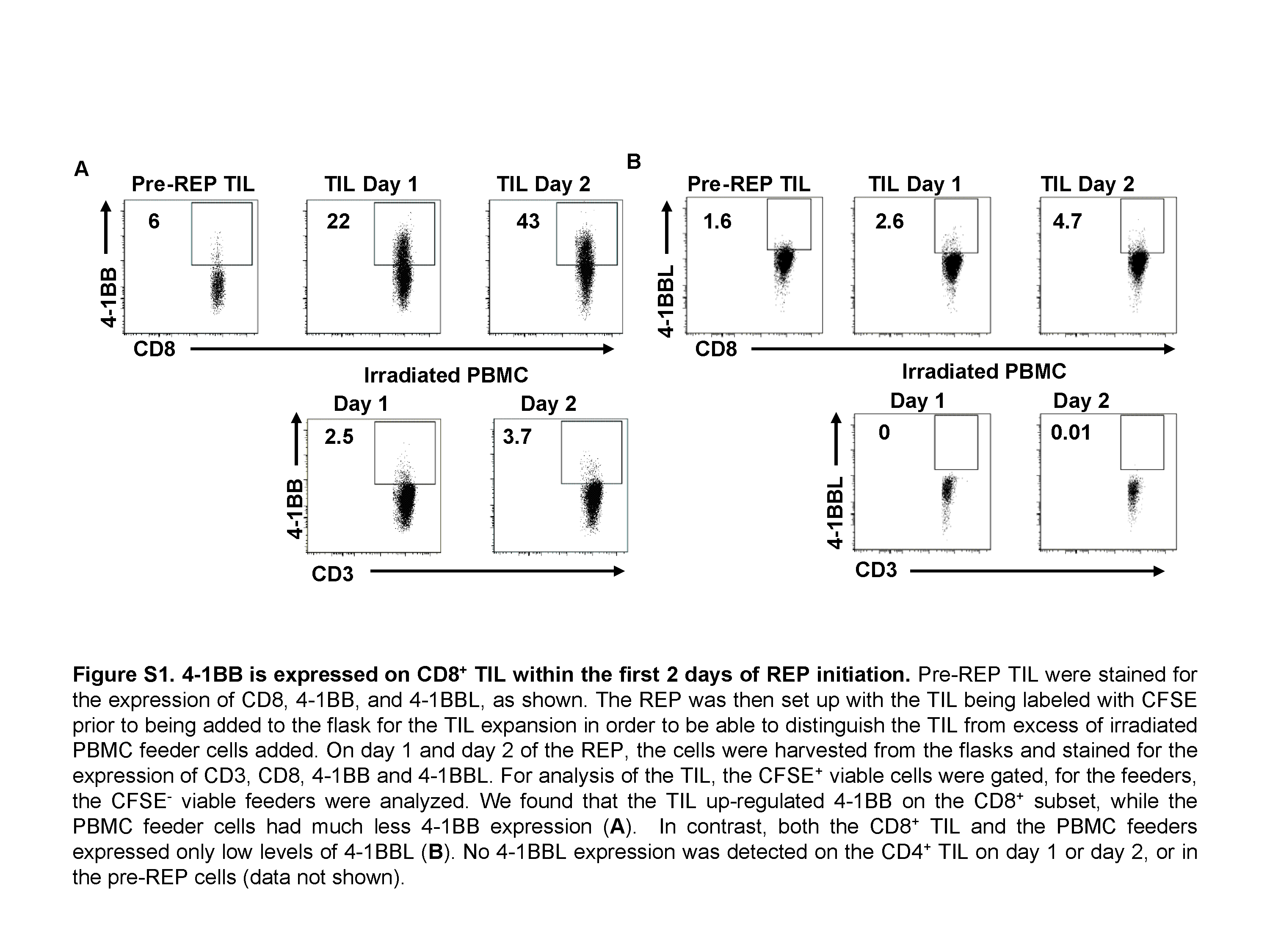

Supplement: Figure S1 — 4-1BB is expressed on CD8+ TIL within the first 2 days of REP initiation. Pre-REP TIL were stained for the expression of CD8, 4-1BB, and 4-1BBL, as shown. The REP was then set up with the TIL being labeled with CFSE prior to being added to the flask for the TIL expansion in order to be able to distinguish the TIL from excess of irradiated PBMC feeder cells added. On day 1 and day 2 of the REP, the cells were harvested from the flasks and stained for the expression of CD3, CD8, 4-1BB and 4-1BBL. For analysis of the TIL, the CFSE+ viable cells were gated, for the feeders, the CFSE- viable feeders were analyzed. We found that the TIL up-regulated 4-1BB on the CD8+ subset, while the PBMC feeder cells had much less 4-1BB expression (A). In contrast, both the CD8+ TIL and the PBMC feeders expressed only low levels of 4-1BBL (B). No 4-1BBL expression was detected on the CD4+ TIL on day 1 or day 2, or in the pre-REP cells (data not shown). (TIF) [file pone.0060031.s001.tif]

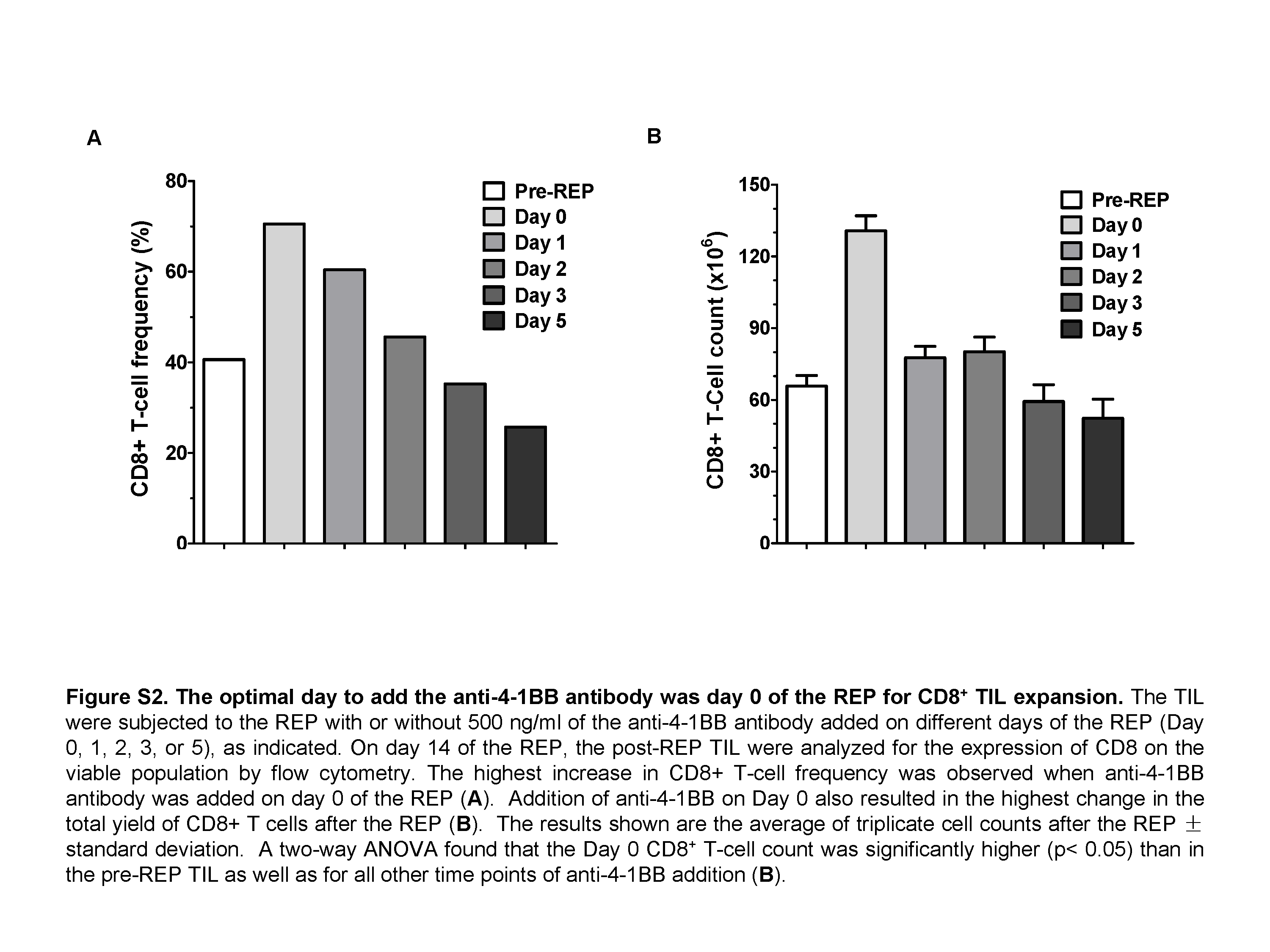

Supplement: Figure S2 — The optimal day to add the anti-4-1BB antibody was day 0 of the REP for CD8+ TIL expansion. The TIL were subjected to the REP with or without 500 ng/ml of the anti-4-1BB antibody added on different days of the REP (Day 0, 1, 2, 3, or 5), as indicated. On day 14 of the REP, the post-REP TIL were analyzed for the expression of CD8 on the viable population by flow cytometry. The highest increase in CD8+ T-cell frequency was observed when anti-4-1BB antibody was added on day 0 of the REP (A). Addition of anti-4-1BB on Day 0 also resulted in the highest change in the total yield of CD8+ T cells after the REP (B). The results shown are the average of triplicate cell counts after the REP ± standard deviation. A two-way ANOVA found that the Day 0 CD8+ T-cell count was significantly higher (p<0.05) than in the pre-REP TIL as well as for all other time points of anti-4-1BB addition (B). (TIF) [file pone.0060031.s002.tif]

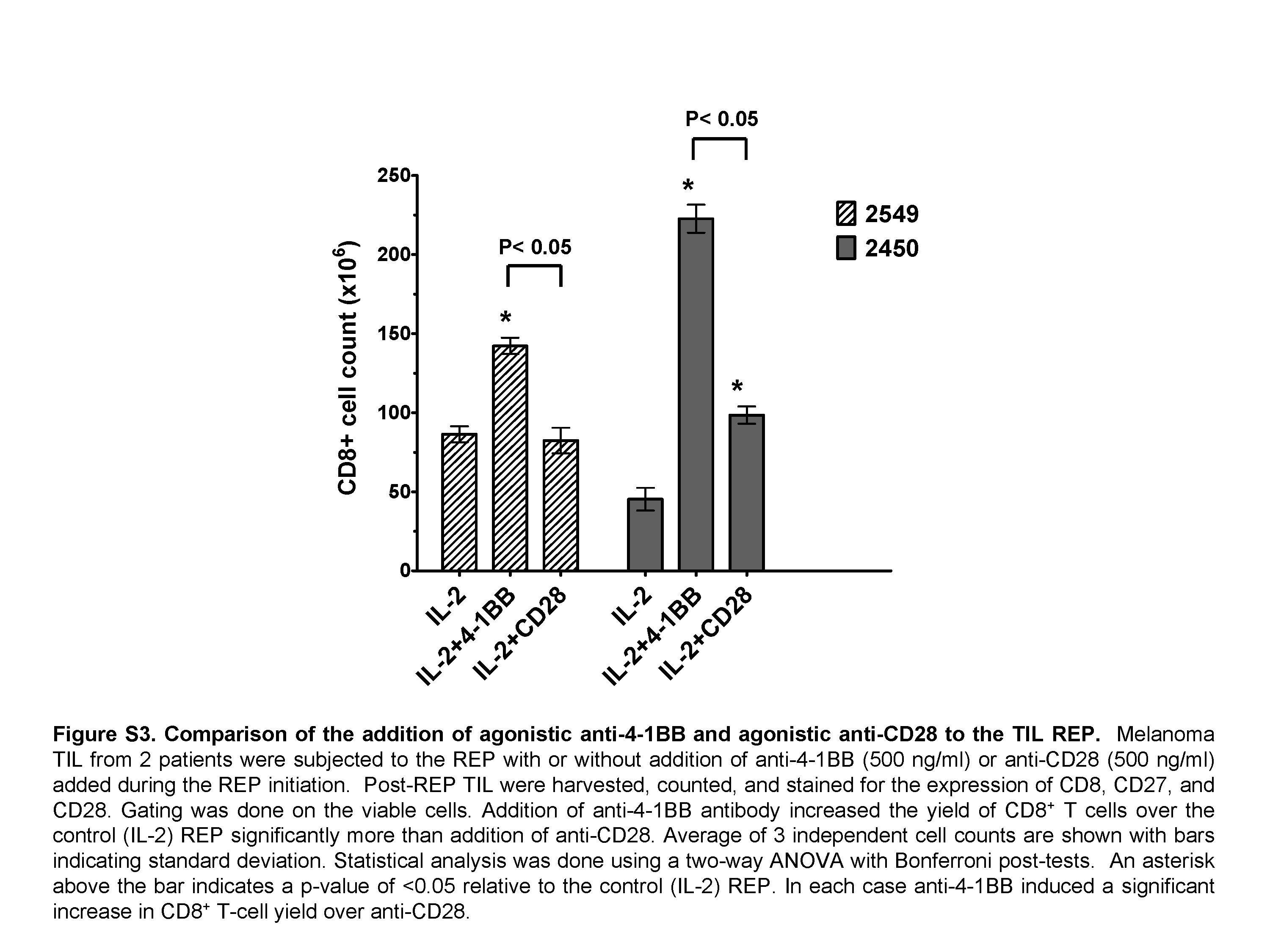

Supplement: Figure S3 — Comparison of the addition of agonistic anti-4-1BB and agonistic anti-CD28 to the TIL REP. Melanoma TIL from 2 patients were subjected to the REP with or without addition of anti-4-1BB (500 ng/ml) or anti-CD28 (500 ng/ml) added during the REP initiation. Post-REP TIL were harvested, counted, and stained for the expression of CD8, CD27, and CD28. Gating was done on the viable cells. Addition of anti-4-1BB antibody increased the yield of CD8+ T cells over the control (IL-2) REP significantly more than addition of anti-CD28. An average of 3 independent cell counts are shown with bars indicating standard deviation. Statistical analysis was done using a two-way ANOVA with Bonferroni post-tests. An asterisk above the bar indicates a p-value of <0.05 relative to the control (IL-2) REP. In each case anti-4-1BB induced a significant increase in CD8+ T-cell yield over anti-CD28. (TIF) [file pone.0060031.s003.tif]

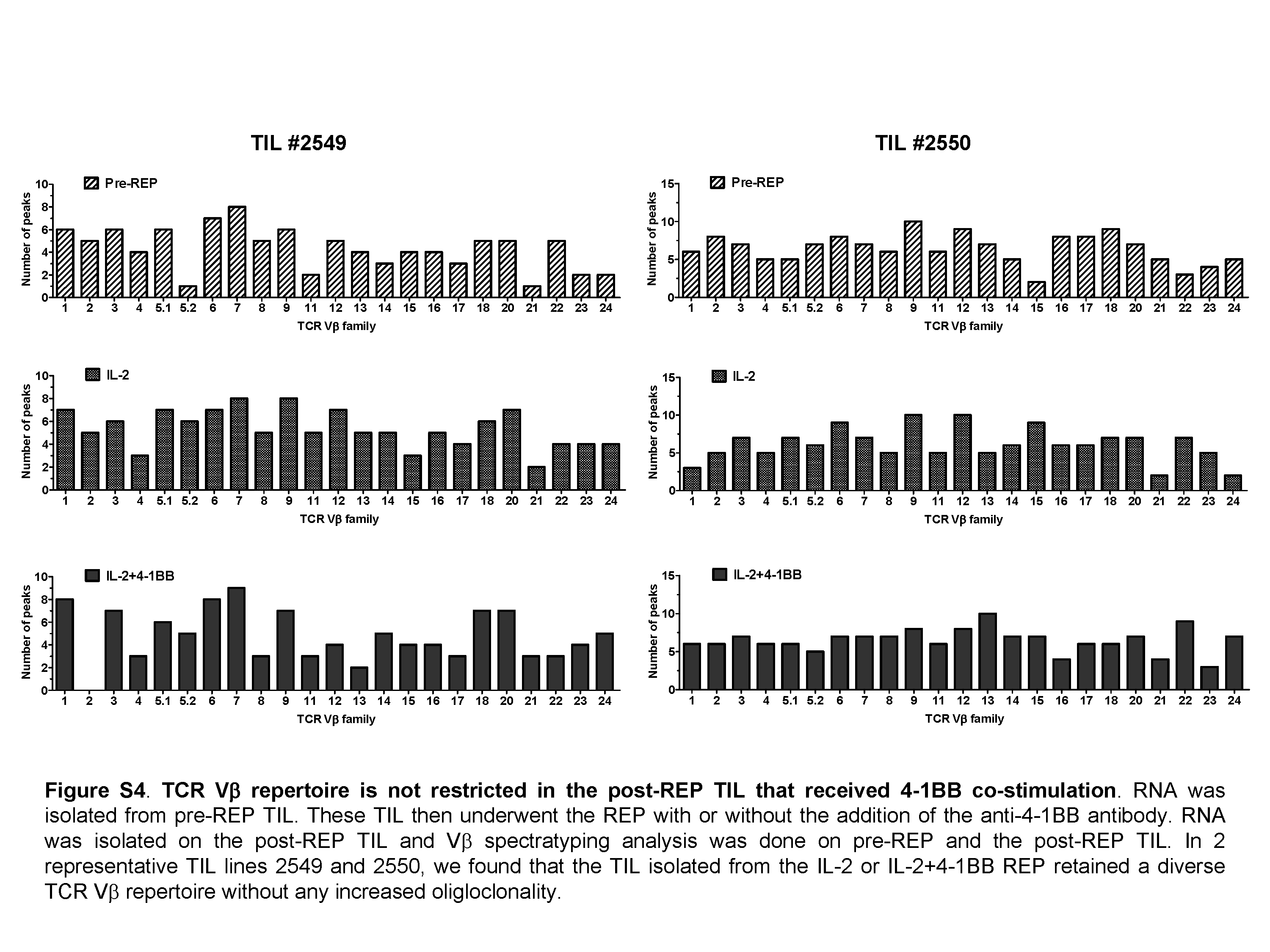

Supplement: Figure S4 — TCR Vβ repertoire is not restricted in the post-REP TIL that received 4-1BB co-stimulation. RNA was isolated from pre-REP TIL. These TIL then underwent the REP with or without the addition of the anti-4-1BB antibody. RNA was isolated on the post-REP TIL and Vβ spectratyping analysis was done on pre-REP and the post-REP TIL. In 2 representative TIL lines 2549 and 2550, we found that the TIL isolated from the IL-2 or IL-2+4-1BB REP retained a diverse TCR Vβ repertoire without any increased oligloclonality. (TIF) [file pone.0060031.s004.tif]

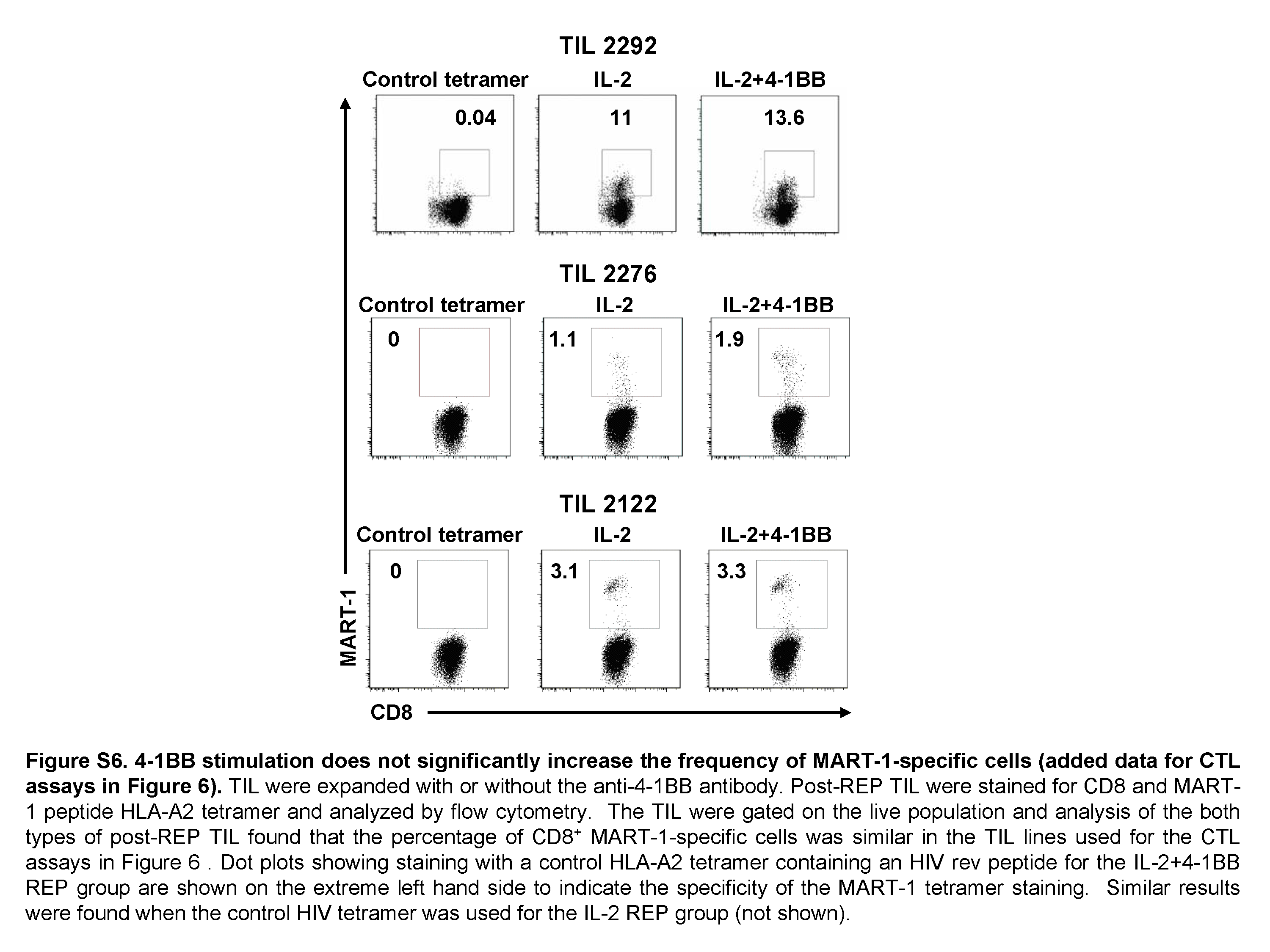

Supplement: Figure S6 — 4-1BB stimulation does not increase the frequency of MART-1-specific cells. TIL were expanded with or without the anti-4-1BB antibody. Post-REP TIL were stained for CD8 and MART-1 tetramer. FACS The TIL were gated on the live population and analysis of the both types of post-REP TIL found that the percentage of CD8+ MART-1-specific cells was similar in 3 representative TIL lines (TIF) [file pone.0060031.s006.tif]
